# PPI-MASS: An Interactive Web Server to Identify Protein-Protein Interactions From Mass Spectrometry-Based Proteomics Data

**DOI:** 10.3389/fmolb.2021.701477

**Published:** 2021-07-01

**Authors:** Mariela González-Avendaño, Simón Zúñiga-Almonacid, Ian Silva, Boris Lavanderos, Felipe Robinson, Roberto Rosales-Rojas, Fabio Durán-Verdugo, Wendy González, Mónica Cáceres, Oscar Cerda, Ariela Vergara-Jaque

**Affiliations:** ^1^ Center for Bioinformatics, Simulation and Modeling (CBSM), Faculty of Engineering, Universidad de Talca, Talca, Chile; ^2^ Millennium Nucleus of Ion Channel-Associated Diseases (MiNICAD), Santiago, Chile; ^3^ Program of Cellular and Molecular Biology, Institute of Biomedical Sciences (ICBM), Faculty of Medicine, Universidad de Chile, Santiago, Chile

**Keywords:** protein-protein interaction, mass spectrometry-based proteomics, PPI-MASS, TRPM4, TMPRSS11A

## Abstract

Mass spectrometry-based proteomics methods are widely used to identify and quantify protein complexes involved in diverse biological processes. Specifically, tandem mass spectrometry methods represent an accurate and sensitive strategy for identifying protein-protein interactions. However, most of these approaches provide only lists of peptide fragments associated with a target protein, without performing further analyses to discriminate physical or functional protein-protein interactions. Here, we present the PPI-MASS web server, which provides an interactive analytics platform to identify protein-protein interactions with pharmacological potential by filtering a large protein set according to different biological features. Starting from a list of proteins detected by MS-based methods, PPI-MASS integrates an automatized pipeline to obtain information of each protein from freely accessible databases. The collected data include protein sequence, functional and structural properties, associated pathologies and drugs, as well as location and expression in human tissues. Based on this information, users can manipulate different filters in the web platform to identify candidate proteins to establish physical contacts with a target protein. Thus, our server offers a simple but powerful tool to detect novel protein-protein interactions, avoiding tedious and time-consuming data postprocessing. To test the web server, we employed the interactome of the TRPM4 and TMPRSS11a proteins as a use case. From these data, protein-protein interactions were identified, which have been validated through biochemical and bioinformatic studies. Accordingly, our web platform provides a comprehensive and complementary tool for identifying protein-protein complexes assisting the future design of associated therapies.

## Introduction

Proteins rarely act as isolated species to perform their biological functions (Berggård et al., 2007). Protein-protein associations regulate diverse molecular and cellular mechanisms in all organisms. Therefore, a critical step towards unraveling structural and functional relationships between proteins is mapping protein-protein physical contacts. A recent reference map of the human interactome revealed approximately 53,000 protein-protein interactions (PPIs) (Luck et al., 2020), indicating an important role in physiological and pathological processes. Numerous human diseases are caused by alterations in the binding interface of protein-protein complexes (Gonzalez and Kann, 2012; Rabbani et al., 2018), making the detection of binary PPIs an essential step for the structure-based therapy design.

Elucidation of PPIs is not trivial as many complexes are involved in diverse biological functions and the component proteins may be interconnected in multiple signaling pathways. Experimental techniques to detect PPIs integrate methods such as yeast two-hybrid (Y2H) screening, fluorescence resonance energy transfer (FRET), atomic force and electron microscopy, surface plasmon resonance, and tandem mass spectrometry (MS) (Berggård et al., 2007; Rao et al., 2014; Peng et al., 2017). MS-based proteomics methods have gained great relevance, offering high sensitivity and specificity to decipher PPI networks and to study their interaction dynamics (Yakubu et al., 2019; Yugandhar et al., 2019). Tandem mass spectrometry (LC-MS/MS) represents a rapid and efficient method for the high-throughput analysis of PPIs, detecting contacts among a group of proteins rather than determining binary complexes (Richards et al., 2021). Typically, the resulting protein data set is over 100–1,000 candidates, making it difficult to distinguish between protein functional associations and protein-protein physical contacts. Efficient and reliable postprocessing computational tools are, therefore, required to identify true-positive PPI partners.

A variety of bioinformatics methods are currently available to identify and quantify peptides and proteins obtained from MS-based proteomics experiments (Chen et al., 2020), providing flat text files with a list of proteins associated with a target protein. Among the identified proteins, non-specific interactors or artifact proteins may be included. Therefore, an exhaustive data analysis is necessary to detect binary protein-protein complexes with the potential to be studied in a pharmacological context. Free access software to assist the analysis of groups of proteins detected by MS-based interactomics experiments include Interactome3D, for the structural annotation and modeling of protein-protein interactions (Mosca et al., 2013); STRING, for mapping all interaction evidence onto a common set of proteins (Szklarczyk et al., 2019); Proteo3Dnet, for generating a structured overview of a set of input proteins in terms of their interaction (Postic et al., 2020); PINA4MS, for identifying interactions between multiple groups of genes incorporating tissue-specific expression data (Wu et al., 2009), among others. Despite the fact that all these algorithms offer a simple and automatized detection of PPI networks, there are no standardized parameters to distinguish novel physical and functional protein-protein interactions. In consequence, MS data are often manually analyzed, identifying binary PPIs according to different physiological properties and functions, which is certainly tedious and time-consuming.

Here, we present an interactive web server called PPI-MASS, which has been designed to identify putative proteins interacting with a target protein. Though a computational pipeline, information is extracted for a defined protein set, integrating sequence and functional data from UniProtKB (The UniProt Consortium, 2021), structural properties from the Protein Data Bank (PDB) (Berman et al., 2000) and the Swiss-Model repository (Schwede et al., 2003), expression profiles from The Human Protein Atlas (Thul and Lindskog, 2018), associated pathologies from DisGeNET (Piñero et al., 2020), as well as drugs modulating the protein function from the DrugCentral (Ursu et al., 2017) and DrugBank (Wishart et al., 2008) databases. Thus, PPI-MASS offers a web platform to guide users in filtering a protein set using different parameters and finding PPIs with pharmacological potential, representing an important starting point for further biochemical studies and computational modeling. The functionality of PPI-MASS has been demonstrated by analyzing the TRPM4 and TMPRSS11a interactomes, identifying key protein-protein binding partners, whose interaction has been validated by means of biochemical assays and molecular modeling. Overall, our web platform provides a simple, effective, and practical tool for the identification of binary protein-protein complexes, which is an essential step for developing PPI-focused drug technologies.

## Implementation of the PPI-MASS Web Server

**FIGURE 1:**
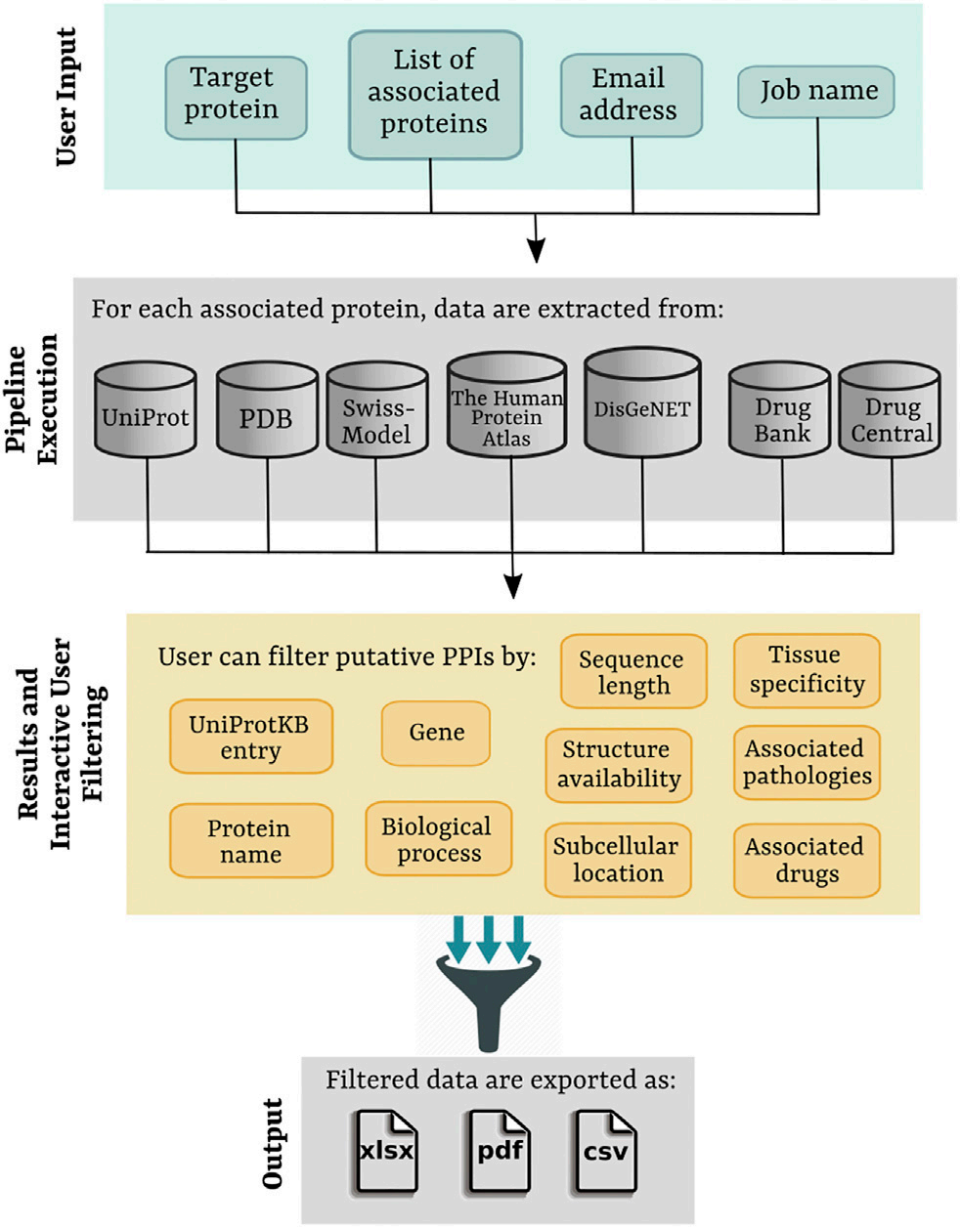
Workflow of the PPI-MASS web server describing the four major layers of the platform to process MS-based proteomics data and to identify candidate proteins to establish PPIs with a target protein.

PPI-MASS is a computational pipeline to obtain biological information of a large protein set, obtained ideally from MS-based proteomics data, in order to identify protein-protein interactions. To make PPI-MASS open access, a web server was developed, which is currently accessible at https://minicad.appsbio.utalca.cl/ppi-mass. The workflow of the web platform is shown in Figure 1, involving the following four layers:

**User Input.** To start its operation, PPI-MASS requires inputs as 1) a target protein specified by its identifier in UniProtKB and 2) a list of proteins associated with the target protein, ideally obtained as a result of MS-based proteomics analyses. The protein set may be entered directly into the text box using their UniProtKB identifiers separated by comma or by uploading a flat-text file with the data separated by line breaks. A 3) job name is necessary to label the processed data, whereas a 4) e-mail address is required to send a link to access the results. Since PPI-MASS does not require any user registration, providing a correct e-mail address to contact the user is highly recommended. An example option to test for proper operation of the web server is provided by pressing “Load example data”.

**Pipeline execution.** Upon submission, the jobs are entered into a queuing system and a universally unique identifier (UUID) is assigned, which may be used later to search the job results. Once ready, the protein set associated with the target protein is analyzed. For each protein, biological information is collected from seven different public databases. The first pipeline step consists in extracting from the UniProtKB database (The UniProt Consortium, 2021) sequence and functional information for each query protein using its UniProtKB identifier. The extracted information includes protein name, gene, biological process, subcellular location, and protein sequence in FASTA format. The number of amino acids making up the protein is calculated from the full-length sequence. Subsequently, availability of structural information is consulted in the UniProt “structure section” cross-referenced to the PDB database (Berman et al., 2000). In the absence of structure, comparative models of the query proteins are searched in the Swiss-Model Repository (Schwede et al., 2003). The PDB files corresponding to the available protein structures or models are analyzed to count the number of amino acids in the primary sequence. A coverage percentage is then calculated as a difference in the number of amino acids contained in the full-length protein sequence. In case that more than one protomer of the protein exists in the PDB file, the coverage of the one with the longest chain is calculated.

To examine the protein expression in human tissue types, “The Tissue Atlas” section in The Human Protein Atlas database (Thul and Lindskog, 2018) is consulted. The gene name obtained from the UniProtKB database is used as identifier for the query. Only human tissues exhibiting high expression levels of the query protein are extracted. Likewise, human pathologies associated with each gene are obtained from the DisGeNET database (Piñero et al., 2020), containing a wide collection of genes and genomic variants associated with human diseases. The section “Summary Gene-Disease Associations” is consulted, and the field “Disease” is extracted. Finally, drugs modulating the function of the query proteins are searched in the DrugCentral (Ursu et al., 2017) and DrugBank (Wishart et al., 2008) databases entering the UniProtKB identifier. All the collected information through the pipeline is integrated into a JSON file, which is managed through the DataTables JQuery plugin to display a result page containing an interactive and user-friendly HTML table.

**FIGURE 2:**
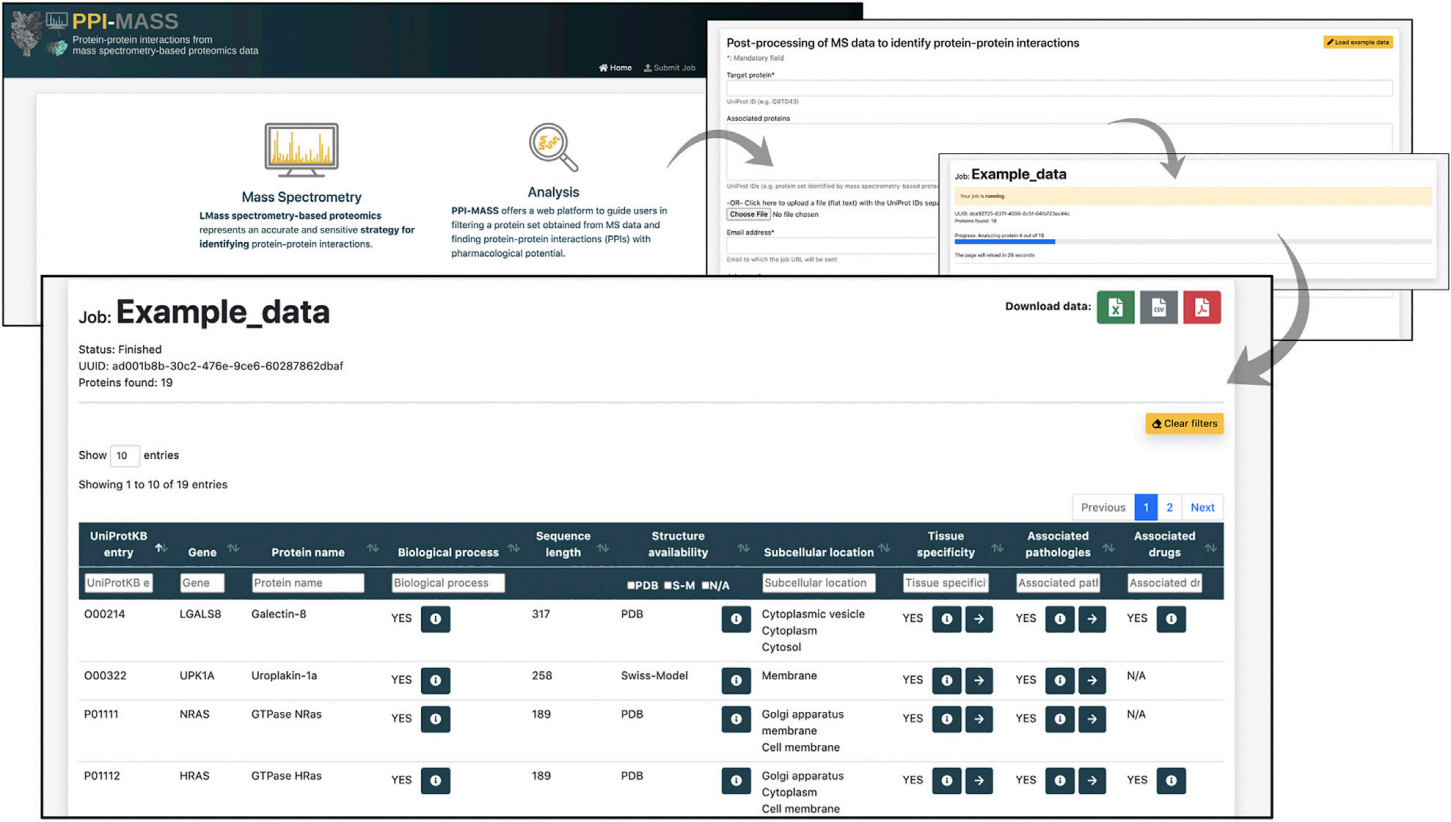
Representative example of a job processed through the PPI-MASS web server. The result page includes an interactive table with ten fields that allow the users filtering a protein set according to different parameters of interest. The buttons allow to expand the results into a modal dialogue box or provide access links to the databases used as reference.

**Results and Interactive user filtering.** Once the job is submitted, and the computational pipeline is executed, the user is automatically directed to the result page. The job’s progress is informed, and an auto-refresh of the page remains active while the job finishes. As previously mentioned, a link to access the results is also sent by email. The resulting data are displayed in an interactive table including ten different fields (Figure 2). The fields 1) UniProtKB entry and 2) Gene correspond to unique identifiers of the analyzed protein set. Section 3) Protein name provides a brief description of the protein name and activity (e.g., Tubulin beta-3 chain, E3 ubiquitin-protein ligase CHIP, Chloride channel CLIC-like protein 1, etc). Similarly, section 4) Biological process describes different processes where the proteins have been involved (e.g., cell adhesion, immune response, viral process, transmembrane transport, etc). Two fields are incorporated to display information about the protein sequence and structure: 5) Sequence length, showing the total number of amino acids in the full-length protein, and 6) Structure availability, reporting experimentally determined structures or models stored in the PDB and Swiss-Model repository. Checkboxes allow the user to toggle PDB codes, Swiss-Model identifiers, or proteins with no available structure. Access links and the coverage of the available structures and models are reported in each case. The location of the mature protein in the cell is informed in section 7) Subcellular location (e.g., membrane, cytoplasm, Golgi apparatus, etc), whereas 8) Tissue specificity indicates human organs where the proteins have shown high expression levels (e.g., colon, lung, thyroid gland, etc). Additionally, diseases associated with the query protein are listed in section 9) Associated pathologies (e.g., carcinogenesis, asthma, cystic fibrosis, etc) and drugs modulating the protein functions are reported in (10) Associated drugs. Links to access the data from The Human Protein Atlas (Thul and Lindskog, 2018), DisGeNET (Piñero et al., 2020), DrugCentral (Ursu et al., 2017) and DrugBank (Wishart et al., 2008) are provided in each case (if applicable). Based on all this information, the users may filter out a protein set according to one or more fields of interest in order to identify putative candidates to establish protein-protein interaction with a target protein. Multiple selections can be tested by applying “Clear filters” to reset the search.

**Output.** The resulting data, including all the filters applied by the user, may be exported to .xlsx, .pdf, or .csv files. The users might also access the contact page to request any assistance in using the web server or visit the user guide to find instructions for the common tasks of the platform.

The PPI-MASS web platform is a freely available prototype. The web server is hosted on a Linux server with an Intel Xeon 2.4 GHz CPU including 4 cores and 6 GB of memory. The web interface was developed with the Python-based web framework Django v3.1, along with the Bootstrap CSS framework v5.0. The computational pipeline was written in Python v3.8.3.

## Case Studies: the Interactome of TRPM4 and TMPRSS11a

To demonstrate the functionality of the PPI-MASS web server, two case studies were analyzed including the interactome of two transmembrane proteins: the Transient Receptor Potential cation channel subfamily M member 4 (TRPM4) and the Transmembrane Serine protease 11A (TMPRSS11a).

**TRPM4.** This protein corresponds to an Ca^2+^-activated non-selective cation channel expressed in several tissues and involved in a variety of physiological processes, including cell migration and contractility (Cáceres et al., 2015). Since TRPM4 is expressed in different brain areas, it has been linked to mental and neurological disorders (Hazalin et al., 2020; Wang et al., 2020). Therefore, understanding the deregulation mechanisms of TRPM4, specifically its increased localization at the plasma membrane, represents an important challenge to intervene in TRPM4-related diseases. To identify TRPM4-associated proteins modulating the channel’s function, our group immunopurified human TRPM4 protein-containing complexes from HEK293 cells and analyzed their components by LC-MS/MS (Cáceres et al., 2015). A set of 124 proteins were identified and processed through the PPI-MASS web server, in order to find putative candidates to interact with TRPM4. For example, and considering the TRPM4 expression in the brain, the filters “cerebellum” or “cerebral cortex” in Tissue specificity were applied, reducing the protein set to 35. Likewise, introducing the filter “mental” in Associated pathologies, 21 proteins were detected. Additionally, to find proteins regulating the expression of TRPM4 at the plasma membrane, the “protein targeting” filter was introduced in Biological process, identifying a total of 4 proteins candidates to establish protein-protein interaction with TRPM4. Interestingly, in this reduced set, the 14-3-3 protein gamma (*γ*) isoform was found, which has been previously reported as a binding partner for TRPM4 affecting its localization at the plasma membrane (Cho et al., 2014). The TRPM4-14-3-3*γ* interaction has been corroborated through co-immunoprecipitation and bimolecular fluorescence complementation (BiFC) assays, demonstrating that both proteins are located in close spatial proximity. In addition, mutations on the S88 residue of TRPM4 revealed a significant reduction in the 14-3-3*γ* binding, indicating a key role of this residue for the protein-protein physical association. Identifying this particular complex validates the functionality of our web platform to detect protein-protein interactions that may be used as therapeutical targets.

**TMPRSS11a.** This protein is a Type II transmembrane serine protease (TTSP) participating in diverse biological processes. As reported recently by our group, a differentiated expression of TMPRSS11a with age was identified in gingival and skin fibroblasts, which has been associated with senescence processes and wound healing (Fernandez et al., 2021). Moreover, it was demonstrated that TMPRSS11a participates in critical cellular responses, including cell migration and spreading. Based on these results, affinity chromatography and MS-based proteomics analyses were carried out in order to identify TMPRSS11a-associated proteins and to decipher the mechanism by which TMPRSS11a might play a role in cell migration. MS assays exhibited 305 endogenous proteins associated with TMPRSS11a, which were processed through the PPI-MASS web server. The “cell migration” filter in Biological process was applied to identify proteins related with TMPRSS11a participating in the same cellular process. The number of candidates was reduced from 305 to 19. Subsequently, taking as a reference the location of TMPRSS11a at the plasma membrane, the “membrane” filter in Subcellular location was used to detect membrane proteins potentially interacting with TMPRSS11a. The protein set was reduced to 11. Computational structural biology studies were then considered to evaluate protein-protein physical contacts. Thus, all proteins with available structures in the Protein Data Bank were selected, decreasing the number of candidates to 8. From this data set, the Integrin beta-1 protein was identified as the most likely candidate to interact with TMPRSS11a. Interestingly, integrins are recognized targets of proteins containing an arginine-glycine-aspartic acid (RGD) motif (Moreno-Layseca et al., 2019), which was identified in TMPRSS11a. Considering this evidence, protein-protein docking, and molecular dynamics simulations were performed, identifying a favored TMPRSS11a:Integrin complex detailed at Fernandez et al., 2021. The physical association of both proteins was predicted to occur between the RGD motif of TMPRSS11a and a pocket located at the top face of the *α*5*β*1 integrin. Proximity ligation assays (PLA) were used to validate close proximity between TMPRSS11a and beta-1 Integrin, whereas immunocytochemistry experiments revealed co-localization of both proteins at the plasma membrane.

## Conclusions and Future Perspectives

Protein-protein interactions play critical roles in different physiological processes of a wide variety of proteins. Therefore, identifying binary protein-protein complexes has become an important approach to study associated diseases. In this regard, the PPI-MASS web server represents a powerful tool for an automatized analysis of MS-based proteomics data, obtaining key information of a protein set associated with a target protein to identify protein-protein interactions with therapeutic potential. As demonstrated in the two case studies presented here, PPI-MASS has been an efficient and useful method to detect PPIs in our group, and it could be of great benefit to the scientific community working in this area.

**FIGURE 3:**
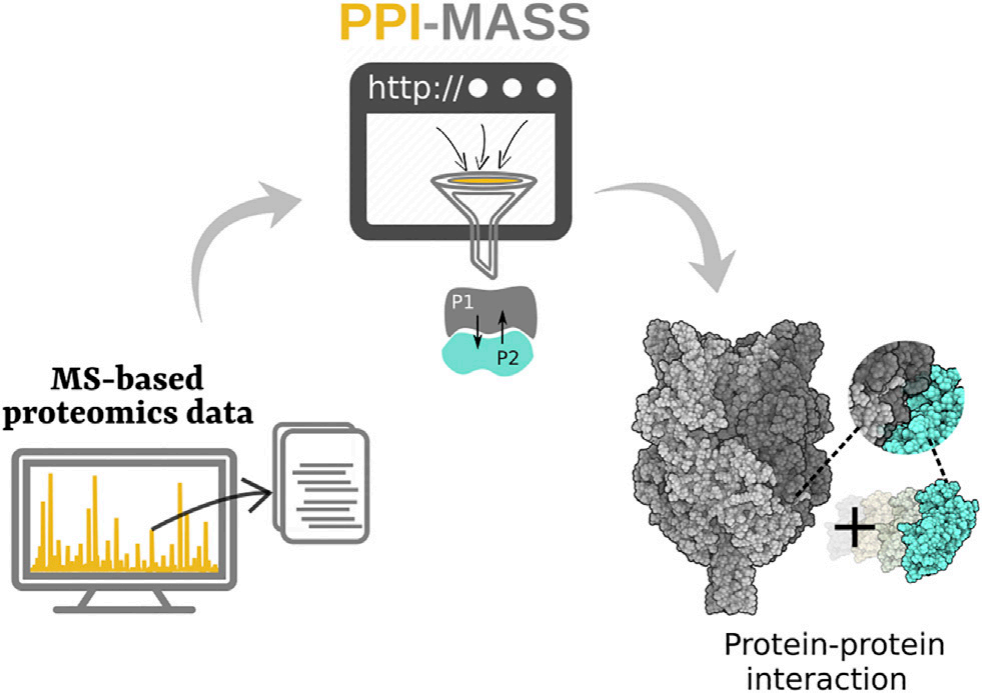
Schematic representation of the PPI-MASS web server acting as a computational interface to process MS-based proteomics data and to predict a structural model of protein-protein interactions.

It should be noted that PPI-MASS is an ongoing project, and further developments will be focused on the incorporation of new parameters to predict protein-protein physical contacts, once a binary complex is identified. As is shown in Figure 3, PPI-MASS is currently a computational platform designed for the post-processing of MS-based proteomics data. However, computational modeling of protein-protein complexes will be integrated in the future to get a deeper insight into their action mechanisms.

## Data Availability

The original contributions presented in the study are included in the article/supplementary material, further inquiries can be directed to the corresponding authors.
